# The role of zinc in the premature brain: functions, outcomes and future research perspectives

**DOI:** 10.3389/fped.2024.1496846

**Published:** 2024-12-23

**Authors:** Myrsini Chamakioti, Luc P. Brion, Pranav Viswanathan, Cheryl S. Lair, Dimitrios Angelis

**Affiliations:** ^1^ Health and Precision Medicine, Choremion Laboratory, Aghia Sophia Children’s Hospital, University Research Institute of Maternal and Child, Athens, Greece; ^2^ Department of Pediatrics, Division of Neonatology, University of Texas Southwestern Medical Center, Dallas, TX, United States; ^3^ Medical School, University of Texas Southwestern Medical Center, Dallas, TX, United States; ^4^ Neonatal Nutrition, Parkland Health, Dallas, TX, United States

**Keywords:** zinc, brain, neonates, neurodevelopment, mechanisms

## Abstract

Zinc (Zn) is one of the most prevalent and essential micronutrients, found in 10% of all human proteins and involved in numerous cellular enzymatic pathways. Zn is important in the neonatal brain, due to its involvement in neurotransmission, synaptic plasticity, and neural signaling. It acts as a neuronal modulator and is highly concentrated in certain brain regions, such as the hippocampus, and the retina. Low Zn intake is frequent in several countries and in populations with high poverty index. Preterm infants are at risk for Zn deficiency for prenatal (missing fetal Zn) and postnatal reasons (less intestinal absorption and insufficient intake in maternal milk to match fetal accretion). The amount of Zn needed for preterm infants is not known and remains the subject of controversy. Recent nutritional recommendations favored an increase in daily Zn supplementation. Systematic reviews of randomized trials have shown that Zn supplementation in preterm infants increases weight gain and may decrease mortality. In this review we will summarize the role of Zn in brain functions and outcomes in preterm newborns, gaps in knowledge and areas of future research.

## Introduction

Zinc (Zn) is intrinsically related to human brain development and function from fetal life to adulthood and is one of the most prevalent micronutrients, involved in numerous cellular enzymatic pathways. In recent years, there is an increasing interest in neonatal research community regarding the correct Zn dose and timing of initiation, Zn level interpretation, effectiveness, and its overall role in improving neonatal outcomes. Zn is considered safe in a wide range of doses, but the actual amount of Zn intake needed to optimize basic cellular functions, growth and neurodevelopmental outcomes, and neonatal morbidities and mortality is not well studied. Recent nutritional recommendations favored an increase in daily Zn supplementation (1–3), supporting the importance of this micronutrient in the well-being of the newborn.

This review is structured in three parts. In part A we describe the roles of Zn in key physiologic and pathophysiologic pathways, enzymes and mechanisms of action related to brain functions as well as data on fetal accretion, neonatal absorption, and homeostasis of Zn. In part B we describe the role of Zn in areas of the brain, neuronal populations and conditions that could be associated with or result from Zn deficiency. In Part C we describe the possible neurotoxic effects of Zn, clinical neurodevelopmental outcomes after Zn supplementation and dosing schedules and rationale in preterm neonates. Although the emphasis is given on preterm newborns, pathophysiologic data are usually derived from animals of other *in vitro* studies and are reported separately.

## Methods

For the literature search, we performed a comprehensive search pertaining to Zn and the nervous system, with emphasis if available on the developing brain. The search involved Pubmed and OVID Medline with the inclusion of a broad variety of terms: “Zn or nervous system,” “brain,” “neurodevelopment,” in combination with the individual search terms for each section of this review such as: “glucose and Zn,” “autoregulation,” “carbonic anhydrase,” “nitric oxide synthase,” etc. Preclinical data are reported separately when available. A summary including controversies and gaps in knowledge is presented in the last part of each paragraph when available, in italic font.

## Part A

### A1 fetal accretion of Zn and neonatal absorption

**Figure 1:**
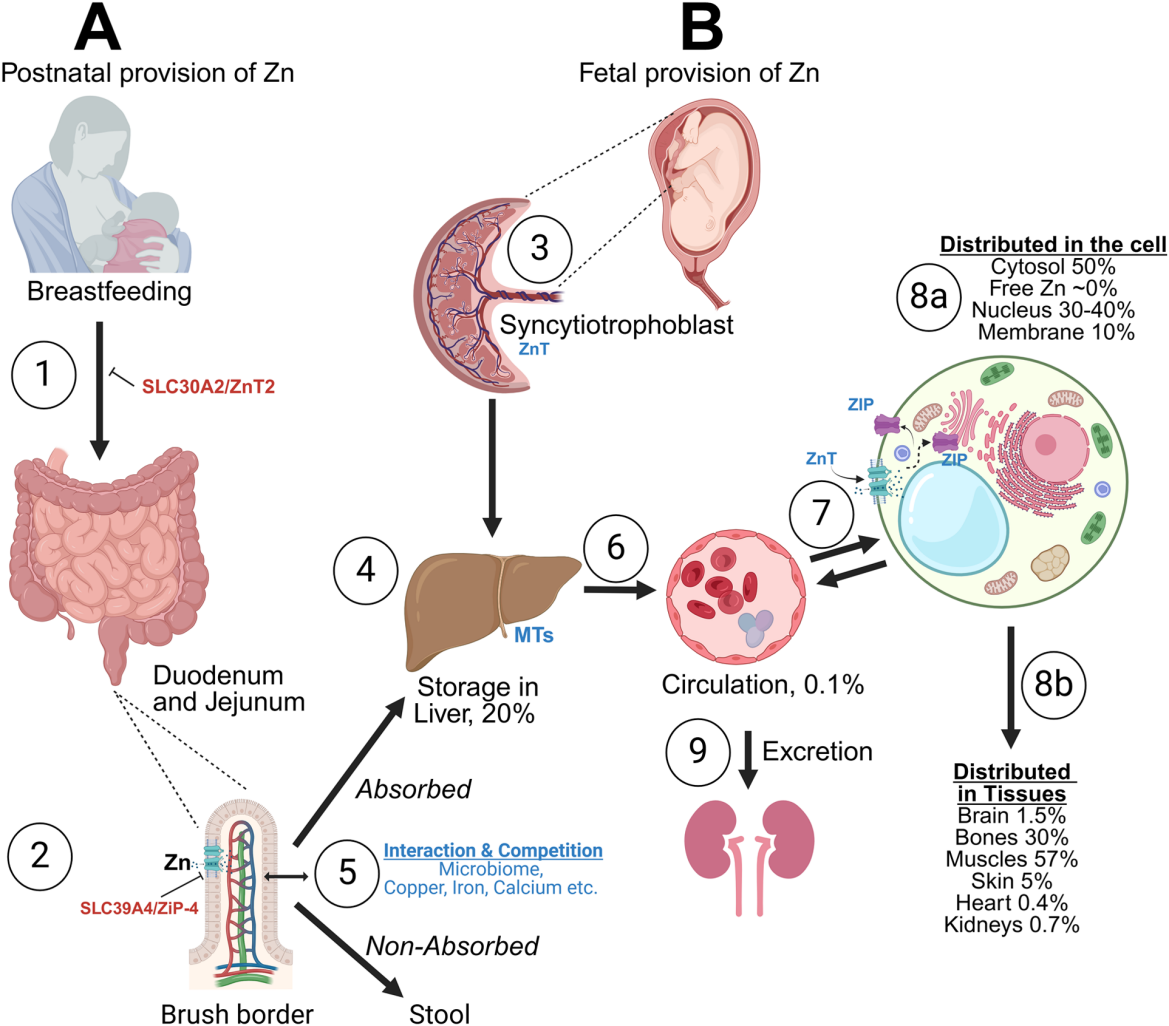
Brief description of perinatal zinc (Zn) transport and storage. **(A)** Zinc (Zn) is provided enterally via human milk or formula and/or intravenously via parenteral nutrition. The absorption occurs with Zn-irk like receptors (ZIP), with competition with other minerals (Cu, Mg, Ca, Fe). Zn transporter 2 mutation can lead to no Zn in human milk (1), while ZIP subtype 4 mutation can prevent Zn absorption in the gut (2). Both conditions can result in severe Zn deficiency. After interactions with the microbiome Zn is absorbed mainly in duodenum and jejunum. About 30% of Zn is absorbed (5). The liver remains the main storage organ, important for Zn homeostasis up to 2 months of life (4). **(B)** In the fetus Zn is transferred to the fetal liver via the placenta. Placenta (syncytiotrophoblast) is important for active transport of Zn against gradients from mother to fetus (3), while the fetal liver is the major storage area throughout the fetal life (4). In circulation (6), Zn is transferred in the red blood cell (RBC) (especially as part of the carbonic anhydrase—CA) and as Zn in serum (mostly bound to proteins, such as albumin, 6). 99% of Zn is intracellular. Interaction at the cellular level and preservation of homeostasis occurs via the receptors Zn transporters (ZnT) (decrease cytoplasmic levels) or ZIP (increase cytoplasmic levels) (7). Intracellularly, Zn is stored in metallothioneins (MT), key proteins for Zn homeostasis. Excess Zn is excreted via the kidneys (9). Intracellular distribution of Zn (8a) and tissue distribution (8b) are shown. Fetal accretion of Zn occurs mainly during the last trimester and hence extreme preterm newborns are at very high risk of Zn deficiency, despite having high serum Zn levels at birth. Created in BioRender.com. Angelis, D. (2024) Agreement number RH27AYOZX5.

The placenta and the fetal liver play a pivotal role in fetal Zn accretion, transfer, and utilization. The proximal bowel is responsible for postnatal accretion. The overall goal is to maintain homeostasis, and steady Zn tissue provision according to the needs of the developing fetus and newborn. The transfer of Zn at the cellular level and among tissues occurs via specific Zn-irk like receptors- ZIP, Solute Carrier family 39A (SLC39A) and receptors Zn transporters (ZnT) (SLC30A), the role of which will be expanded later in this review (Figure 1).

#### Transplacental transport

The syncytiotrophoblast is responsible for Zn uptake from the maternal circulation and the subsequent release into the fetal circulation. The placenta has abilities to adjust the absorption of Zn not only during the progression of gestation (high uptake in premature vs. term vesicles), which supports the needs of the growing fetus, but also in conditions of low maternal Zn consumption or deficiency, which may limit fetal Zn deficiency (4). Zn accretion in mg/day increases progressively from 24 to 36 weeks' gestation but accretion in mg/kg/day (factored for fetal weight) decreases from 24 to 30 weeks and then remains constant (5). Both the Zn importers (ZIP, SLC39) and Zn exporters (ZnT, SLC30) are expressed in the placental syncytiotrophoblast, but the processes that facilitate Zn absorption and especially the mechanisms that control the adaptation in absorption of Zn are not well studied in humans (6).

Early studies in artificially perfused human placental lobules, found that tissue Zn concentration was 10 times higher than the concentrations of perfused (plasma) Zn and suggested that transfer of Zn in the syncytiotrophoblast is active, while transfer towards fetal circulation is passive, via simple diffusion (7). The active transport of Zn against gradients from maternal to fetal circulation is supported by several studies that reported higher Zn level in umbilical vein (UV) when compared to maternal blood (8–11). One mechanism that was recently proposed and can explain how Zn is transferred from the maternal circulation to the placenta against gradients is endocytosis via micro-vesicles. These exhibit saturable characteristics, have a biphasic response (initially rapid and later slow phase of accretion) and depend on potassium gradients (voltage gate properties) (7, 12).

Interestingly, total fetal Zn (bound and free) in term newborns, as measured in UV via atomic spectrometry, was found to be higher when compared to maternal or amniotic Zn, but free Zn was not significantly different (13), and Zn variations were attributed to differences in binding of Zn in plasma proteins. Pregnant women with high serum Zn levels >10.7 μmol/L (0.7 μg/ml) were found to have a higher percentage of Zn bound to alpha 2-macroglobulin compared to women with lower Zn levels, while they also had more bound Zn in albumin in their cord blood (70%) when compared to serum (56%) (4). The differences of protein binding of Zn and their potential effects on placenta transfer need further investigation. Mixed models of Zn transfer (passive and active) have also been suggested in experiments with human perfused placentas, where transport fractions of Zn averaged 0.21% of maternal loading concentration (14).

The factors that influence the transfer of Zn via the placenta are also not well understood. The serum fetal Zn level does not appear to significantly affect the transplacental transfer from the mother to the fetus when *in situ* perfused placentas from guinea pigs are utilized (15, 16). UV concentration was higher than maternal plasma levels, while the maternal to fetal transfer of Zn was directly related to maternal plasma Zn concentrations as well as blood flow in the uterine and umbilical vessels (16).

#### Zn placenta absorption when mother is Zn deficient

Zn deficiency worldwide is common and is encountered more frequently in lower income countries. In the US, Zn deficiency is less common but lower Zn intake could be encountered in mothers with low poverty index, and in women of Mexican origin (17).

Myers et al. described that nutritional content of Zn are expected to deteriorate with rising atmospheric carbon dioxide levels (CO_2_). More than 2 billion people live in countries that receive at least 70% of iron (Fe) and Zn from C_3_ crops (in which photosynthesis starts with 3 carbons, e.g., soybean, rice). Most temperate, non-legume C_3_ crops are unable to extract sufficient nitrogen (N) from soil at high ambient CO_2_ to maintain tissue carbon (C): N ratio. Rising ambient CO_2_ is likely to yield C_3_ crops with less proteins, Fe and Zn content (18).

Zn deficiency is also encountered in high income societies, as recently noted in Japan, where ∼33% of all women and ∼20% of those in reproductive age had Zn levels <0.6 μg/ml (19). In a Japanese study that included mothers with high baseline Zn deficiency, the ratio of UV to maternal plasma Zn was found to be ∼2:1 and there was no difference in normally grown neonates vs. those with intrauterine growth restriction (IUGR), while umbilical arterial (UA) to UV Zn was <1 in normally grown neonates and ∼1 in IUGR (20). There is evidence in both animal and human studies that placenta can adjust Zn absorption when maternal Zn deficiency is present. For example in Zn deprived pregnant mice, oral provision of radioactive Zn in the last part of pregnancy, resulted in higher total fetal body Zn retention, than those with had Zn rich diets (21). In the previously mentioned study, pregnant women with serum Zn >0.7 μg/ml had higher UV Zn when compared with those with lower levels (4). In a randomized controlled trial (RCT), in Gambian pregnant women who received diets poor in Zn, women in the control group had significantly higher mRNA concentrations of placental Zn transporters, when compared to those who were randomized to Zn supplementation (22). In one study in mice IUGR occurred in association with maternal Zn deficiency despite regulation of placenta Zn transporters at mRNA and protein levels, suggesting that regulation of placenta transporters may be insufficient to prevent fetal Zn deficiency (23).

#### Role of liver in Zn storage and usage

The fetal liver can retain large quantities of Zn during fetal life (Figure 1B), accounting for approximately 25% of total body Zn content, in contrast to placenta which operates only as a transient storage area. In rodents, placenta retention of radioactive Zn is limited. Immediately after Zn provision, Zn very quickly distributes from the placenta (high at 2 h, minimal at 24 h) to various tissues especially the fetal liver (quadrupled at 24 h after injection) (24). In humans, liver Zn concentration peaks is about 200–1,020 μg/g at 22–30 weeks' gestation and decreases to 140–380 μg/g at term in countries without endemic Zn deficiency (5, 25). In contrast, fetal liver Zn in Brazil, where Zn deficiency is endemic, is 30–304 μg/g at 26–34 weeks gestation and 13–268 μg/g at 40–41 weeks (26). The role of the fetal/neonatal liver as a major storage area for Zn is maintained for up to the first few months postnatal, assuring Zn homeostasis, despite poor Zn provision through enteral nutrition (27).

#### Postnatal Zn homeostasis

After birth, to meet nutritional needs, the newborn must receive Zn either via mother's own milk (MoM), donor human milk (DHM) or formula and/or via parenteral nutrition including trace elements (PN). In Figure 1, we depict the routes of Zn transfer and storage. Zn is transferred into human milk via the receptor Zn transporter 2 (ZnT2) and absorbed in the duodenum and jejunum via ZIP subtype 4. ZnT2 mutation results in transient Zn deficiency in exclusively breastfed infants, while ZIP4 mutation results in a lifelong disorder, acrodermatitis enteropathica (28). In circulation, Zn is transferred into the red blood cell (RBC) [especially as part of the carbonic anhydrase (CA) system] and as Zn in serum, bound to albumin and other proteins (29). It is likely that Zn is transported across cell membranes via its receptors as free Zn, after release from its ligands, although the exact mechanisms are unknown (6).

Absorption of Zn occurs in the brush border of intestinal mucosa especially in the duodenum and jejunum (30). Differentiation of the enterocyte border and effective length of the jejunum increases with GA (31). For the above reasons, bioavailability of enteral Zn in preterm newborns is only 10%−30%; therefore, enteral Zn needs in preterm infants are estimated as minimum of 4–5 mg/kg/day to match fetal accretion (32–35). Zn content in MoM after term delivery decreases postpartum from a content of 8–12 mg/L in colostrum to 0.7 to 1.6 mg/L by 1 month (36, 37). Zn content in MoM after preterm delivery ranges between 3 and 10 mg/ml in 2 studies (38, 39). The Zn content in DHM (2.14 ± 0.73 mg/L in 11 DHM samples) (40)—which is recommended for preterm infants when the amount of MoM is insufficient- is lower than in preterm MoM, but similar to mature term MoM. Fortification with human milk fortifiers and preterm formula provides about 2 mg/kg Zn per day, thus less than what is needed to match *in utero* accretion (41).

The total intracellular concentration of Zn is high, reaching 200 μM (42), while the free intracellular Zn is in the femtomolar range showing the tight sequestration of intracellular Zn (43). The interaction of Zn at the cellular level and preservation of strict cellular homeostasis occurs via the aforementioned specialized transporters, specifically, ZIP, SLC39A (which increase cytoplasmic Zn levels) and ZnT (SLC30A) (which decrease cytoplasmic Zn levels) (44, 45). Hormones, cytokines, and the availability of Zn dynamically control the subcellular localization and expression of Zn transporters. The highest tissue concentration, beyond the liver, is in bones and pancreas (200 μg/g), compared with most other organs (1–23 µg/g) (29, 44). Areas of the brain with high Zn content include the olfactory apparatus, the cerebrum and hippocampi, while cerebellum has the lowest (46). About 99% of the total body Zn is intracellular. Inside the cell, Zn is distributed between the membrane (10%), the nucleus (30%–40%), and the cytoplasm (50%) (29) (Figure 1).

#### Metallothioneins

Most Zn is stored transiently in specific proteins (metallothioneins, MTs). MTs bind 20% of intracellular Zn as their cysteine sulfur groups create two Zn-sulfur complexes, which can bind up to 7 Zn ions per protein (47, 48). The MT family of proteins includes four isoforms, designated as MT I–IV, with I and II being ubiquitous in all tissues and III being present in central nervous system (CNS), while IV is less well described. The hepatocyte has high content of MTs I and II. Zn can be released from liver MTs upon net deficit, contributing to its homeostasis (25, 49). MTs concentration peaks between 14 and 23 weeks of GA (50) and subsequently decreases with gestation, but the total Zn tissue content is maintained (27). In mammals, MT I expression is downregulated by the increasing maternal estrogen (51). In rodents, placental MTs do not seem to play a significant physiolologic role in binding and transferring of Zn (24). However, induction of placenta MTs after toxic metal exposure (e.g., cadmium) or very high Zn concentrations shows that MTs might play secondary protective roles as will be described later in this chapter (52).

In summary: The placental transfer of Zn from maternal blood to fetal blood and subsequently to the fetal tissues likely occurs against gradient at the syncytiotrophoblast. The fetal liver is a key organ for storage of Zn and maintains this role in the first few months of life, until adequate Zn intake is established. Specific brain areas have very high Zn content. Preterm infants are at risk for Zn deficiency for prenatal (missing Zn accretion during the second and third trimesters) and postnatal reasons (less absorption in the proximal intestine and insufficient Zn even in fortified MoM and DHM to match fetal accretion).

### A2 how do we assess Zn status in preterm newborns?

Measurement of the level of serum Zn, despite representing a subfraction (∼0.1%) of the total Zn pool, is considered the gold standard for monitoring its deficiency. The accretion of Zn occurs mainly during the second and third trimesters and hence extreme preterm newborns are at high risk of Zn deficiency, despite having high serum Zn levels at birth (53). The American Society for Parenteral and Enteral Nutrition (ASPEN) recommends a normal range for Zn concentration of 0.74–1.46 μg/ml (54). Normal serum Zn levels decrease with postnatal age (32, 55–57).

Several investigators including our group have shown that levels <0.7–0.74 μg/ml (deficiency) are associated with abnormal outcomes [poor growth, retinopathy of prematurity (ROP) etc.] (58, 59). The upper limit of normal (>1.46 μg/ml) has not been validated in large studies. UA and UV Zn levels have been utilized to estimate fetal Zn content and as explained earlier UV Zn levels are higher than UA levels, do not always correlate with maternal Zn levels and are affected by conditions associated with the birthing process (60). In a systematic review, cord Zn levels were found to be lower in pregnancies with various pathological outcomes [small for GA (SGA), IUGR, preeclampsia, smoking, etc.] and to correlate with maternal Zn levels (*R* = 0.4365) (61).

Zn concentration in hair could be valuable since it correlates with maternal Zn stores (62); however, obtaining enough sample size for Zn measurement may be a challenge in very preterm newborns. Zn supplementation in preterm newborns increases serum levels (total and skeletal subfraction) of alkaline phosphatase (ALP) (63, 64), a Zn-dependent enzyme. Bone alkaline phosphatase derives mainly from osteoblasts and plays a critical role in bone mineralization (65, 66). Zn deficiency can be suspected in infants with low serum concentration of ALP in the absence of rickets (67). Interestingly, Zn increased the activity and the half-life of ALP *in vitro* (68), without interfering with transcription of ALP protein. These findings could provide a link between Zn, ALP, growth and bone mineralization. It would be compelling to use ALP as an indirect, surrogate method to monitor Zn status since ALP levels are low in association with Zn deficiency and increase with Zn supplementation. However, this method is unlikely to be effective in preterm infants, since ALP may decrease with Zn deficiency and increase with cholestasis, vitamin D deficiency or fractures (63, 64). This may explain why studies in preterm infants have not shown a correlation between serum Zn levels and ALP (66, 69, 70). In addition, Zn deficiency can be encountered more frequently when DHM is provided to preterm neonates (40).

In summary: Measurement of serum Zn level (with normative values: 0.74–1.46 μg/ml) remains the most common and gold standard method for evaluation of Zn, despite its limitations. Serum Zn levels are affected by physiologic factors (GA, postmenstrual age, PMA, birthing process, growth etc.), but also from pathologic factors (inflammation, infection etc.). Umbilical serum Zn level should be used with caution (differences between UA, UV, not well standardized levels, influenced by birthing process etc.). Tissue Zn is ideal but difficult to obtain in neonates.

### A3 mechanisms, enzymatic pathways and biochemical effects of Zn

Zn is an important micronutrient that binds up to 10% of the proteins in the body and operates as a cofactor in a variety of enzymes (45). Zn interferes with or is structural part of enzymes and signaling pathways that as a net effect facilitate euglycemia, cellular cross talk, growth, brain functions and development. In these subsequent sections we summarize the evidence of specific enzymatic and biochemical pathways that could explain some of the benefits of Zn supplementation and some of the potential harms. The vast part of this evidence arises from preclinical animal studies or cell cultures.

#### Nitric oxide (NO) synthase (NOS) pathway

NO pathway dysregulation is important cause of endothelial dysfunction and vascular disease (71, 72). Zn is a part of the structure of all three NOS isoforms (inducible, iNOS, endothelial, eNOS and neuronal, nNOS) (73, 74) and a crucial regulator of the production and activity of these enzymes. The inducible NOS is related to neuroinflammation. Zn and NO operate in a feedback loop. NO produced by iNOS nitrosates Zn-containing intracellular proteins, such as MT. It increases the levels of labile Zn by freeing bound Zn (75). The increased Zn in turn, inhibits the iNOS, reducing NO production and protecting the cells from NO-induced endothelial damage (76).

The mechanism by which Zn inhibits iNOS is by limiting the nuclear factor (NF)-κB transactivation activity. This has been shown by a decrease in the activity of NF-κB-driven luciferase reporter and the NF-κB target genes expression, such as interleukin (IL)-1β and cyclooxygenase (COX) 2. Another mechanism of Zn-mediated inhibition of iNOS is the inhibition of the cytokine-induced activation of the iNOS promoter. Apart from these, Zn facilitates the activity of MTs, which covalently bind NO to form S-nitrothiols, thereby scavenging the cytotoxic NO (77). Zn is also essential for the formation and function of the eNOS in the dimeric—active—form (78). Zn deficiency in the fetal period might cause decreased expression of eNOS in rats (79).

In summary: By inhibiting the NF-κΒ activity, Zn limits iNOS expression, acting as a cytoprotective element against NO-induced inflammation. In addition, Zn deficiency decreases expression of eNOS, a key enzyme for brain autoregulation. The role of Zn as part of the nNOS is not well understood.

#### Carbonic anhydrase (CA)

CA is the first discovered Zn-containing metalloenzyme. It is one of the most catalytically efficient enzymes. Its prominent role is catalyzing the carbon dioxide (CO_2_) conversion into bicarbonate (HCO_3_^−^) and water. CA is abundant across all kingdoms of life (80, 81). CA type II (CAII), the predominant isoform expressed in RBCs, mediates the transport of CO_2_, playing a crucial role in respiration. CA (including several isoforms) is expressed in several tissues including the placenta, kidney, liver, brain, gut and bone, mediates CO_2_/HCO3^−^ equilibrium and blood and tissue acid-base balance (82).

In the brain, CA is expressed in the choroid plexus, oligodendrocytes, myelin, glial cells, and several specialized cells (83). CA has a role in production of cerebrospinal fluid (CSF), in regulation of cerebral blood flow (CBF) (84) and in brain electric activity (85).

CA comprises an active site with a Zn-binding site, an entrance conduit, and various hydrophobic and hydrophilic parts (86). Zn has a vital role in CA catalytic function. More specifically, Zn-bound hydroxide creates Zn-bound bicarbonate by reacting with the carbonyl carbon of CO_2_. As a next step, the Zn-bound bicarbonate is displaced with water. The Zn-bound water releases H^+^, which is transported to the external buffer to regenerate the Zn-bound hydroxide (87). Alternative transition metal ions, like Ni^2+^ and Mn^2+^, can replace Zn. However, this drastically decreases CA catalytic activity, rendering it sometimes completely inactive (88).

Because of the high turnover rate of CA activity, a significant change in its concentration needs to occur before any clinical effect is observed. Severe Zn deficiency in animals decreases the amount of CA protein and CA activity in RBCs and has been associated with tachypnea and even gasping in pullets and rats (89). Zn deficiency decreases CA activity in submandibular gland, tongue epithelium as well as trigeminal and chorda tympani response to carbonated water in rats (90–92). In adults, Zn deficiency decreases RBC CA activity, maximum exercise capacity and taste (93, 94). Zn supplementation improves taste in adults with CA VI deficiency (95).

In summary: Zn is a key component of CA. Severe Zn deficiency can result in decreased CA activity and overt symptomatology in animal studies and in limited human studies, and include fatigue, decreased muscle strength and cardiopulmonary effects. Zn deficiency can also affect the neuronal CA but these effects remain to be investigated.

#### Transcription factors

Zn-finger proteins (ZFPs) are nuclear transcription factors. Like other transcription factor families, they regulate gene expression and affect cell proliferation, differentiation, and cell death (96). ZFPs are DNA-binding domains arranged in various formations. They can read various DNA sequences and use their kinase-binding domains to participate in protein-protein interactions and signaling pathways. In addition, ZFPs play a role in further specialized processes, such as chromatin remodeling, cytoskeleton organization, epithelial development, mRNA trafficking, and cell adhesion (97).

While ZFPs differ in structure, it is widely accepted that in a ZFP, a specific combination of cysteines and histidine amino acid residues chelates a Zn ion, creating a complex that consolidates the domain's 3D structure. The identity and spacing of the Zn-binding amino acids determine the type and specificity of each ZFP (98).

In archetypal DNA binding ZFPs, such as transcription factor IIIA (TFIIIA) and GATA-binding factor 1 (GATA-1, which binds the DNA sequence “GATA”), we see that a beta-hairpin and an alpha-helix are folded around a Zn ion and that it is the alpha-helix that binds with the major groove of DNA. In this way, Zn facilitates the three-dimensional (3D) conformation of these transcription factors. The 3D structures of the ZFP in TFIIIA and GATA-1 are crucial for their DNA-binding and regulatory functions. In both cases, the spatial configuration of these ZFPs allows for high specificity in DNA recognition, influencing the regulation of gene expression. Specifically, the alpha-helix of the ZFP forms hydrogen bonds with three DNA bases of the guanine-rich strand of the DNA major groove. This way, the transcription factor can loop around the oligonucleotide sequence in one turn (99).

In summary: Zn homeostasis is important for the function of transcription factors (such as TFIIIA and GATA-1), facilitating their 3D conformation and their highly selective binding in the DNA grooves, assuring appropriate and tightly regulated gene expression. In this way, Zn can control important processes of gene expression and the following downstream effects.

### A4 immunity

Zn plays a critical role in the immune system. Acute dysregulation of Zn levels hinders the proliferation, maturation, and activation of cells in innate and adaptive immune responses, while chronically disrupted Zn homeostasis increases the risk of inflammation and disease (100, 101). Inflammation (fetal or neonatal) can affect fetal brain development as it relates with abnormal white matter growth and development of periventricular leukomalacia (PVL) (102, 103).

#### Innate immunity

Zn controls various aspects of the innate immune response. *In vitro* extremely high Zn levels (500 μM) induce polymorphonuclear leukocytes (PMN) chemotaxis while very low Zn levels decrease PMN chemotaxis (104). Cell cultures with human cells show variable effects of Zn on cytokines depending on its concentration and cell status (105, 106). Zn overall increases interferon (IFN)-γ, IL-10, IL-1β and tumor necrosis factor (TNF)-α in lipopolysaccharides (LPS)-stimulated cells, while Zn down-regulated levels of IL-1β and TNF-α in peripheral blood mononuclear cells (PBMC) when stimulated with superantigens (107).

Zn, through proteins like the early endosome antigen 1 (EEA1), affects phagocytosis. During sickness and stress serum Zn rapidly declines due to rapid redistribution into the cells, which can be used for protein synthesis as antioxidant and as anti-microbial. Cytokines, such as IL-6 and TNF-α appear to contribute to this physiologic phenomenon (108, 109). Under conditions of Zn deficiency, LPS stimulated mononuclear cells produced higher levels of IL-1β (110, 111). Similar results were shown for TNF-α (111).

Zn excess promotes phagocytes' activity, whereas Zn deficiency has the opposite effect. As a next step after phagocytosis, Zn plays a role in the neutralization of pathogens; abnormal Zn levels, either low or high, inhibit the nicotinamide adenine dinucleotide phosphate oxidases (NADPH). NADPH is crucial for destroying pathogens after phagocytosis, as it controls the production of superoxide anions (112). Regarding monocytes, Zn facilitates their adhesion to the endothelium of the vessels and participates in the production of the pro-inflammatory IL-1β, IL-6, and TNF-α. Zn promotes the expression of ZFPs with anti-inflammatory properties, such as A20, which hinders the activity of NF-κΒ and NF-κΒ target genes, such as IL-1β and TNF, and thus prevents TNF-induced programmed cell death (113, 114). Lastly, Zn is a key element in dendritic cells' maturation process. Downregulation of ZIP-6 decreases the intracellular Zn levels, which subsequently affects the maturation process of dendritic cells and the further activation of the adaptive immune system.

#### Adaptive immunity

T cell progenitors mature in the thymus, and Zn deficiency causes thymic atrophy and T cell lymphopenia. During maturation, pre-T cells are the most susceptible to Zn deficiency, which can lead to a loss of 50% of them in mice (115, 116). Zn is crucial for the adaptive immune response and, most of all, for the development and function of the T-cells. Zn is a co-factor for the activity of the hormone thymulin, which regulates T-cell differentiation and function (47, 117). Moreover, in activated T-cells, Zn is required for signal transmission during IL-2-induced proliferation (118). Zn affects the TH1/TH2 balance. In the case of Zn deficiency, TH1 cytokines such as IFN-γ, IL-2, and TNF- α are reduced. However, the production of TH2 interleukins, like IL-4, IL-6, and IL-10, does not change. This results in an imbalance between TH1 and TH2 interleukin levels, which Zn supplementation restores (119, 120). Lastly, in cases of Zn deficiency, the levels of glucocorticoid hormones are elevated. The anti-apoptotic protein Bcl-2 is also reduced. This combination promotes pre-T cell apoptosis (115, 121).

Although B cells are much less affected by Zn levels than T cells, Zn is necessary for the survival of premature B cells and antibody production and, thus, crucial for the B-cell antigen-specific immune response (47, 122).

In summary: Modulation of immunity under conditions of Zn deficiency could impact neuronal functions via the direct impact on neuroinflammation. Unfortunately, clinical evidence is lacking so far.

### A5 metallothioneins (MTs) and the brain

MTs can act as Zn acceptors and donors inside the cell, exchanging metal ions with proteins. MTs control the intracellular Zn levels with their sulfur cysteine groups, which release Zn after undergoing oxidation (123).

MTs provide age-dependent protection against neuronal toxicity with higher protective effect with advancing age as shown in rodents (124). Oxidizing conditions promote the release of Zn while reducing conditions restore Zn binding to MTs (125). By upregulating the expression of the metal regulatory transcription factor 1 (MTF-1), Zn increases the synthesis of MTs (126). MTs, in turn, act as metal scavengers and prevent the Fenton reaction and reactive oxygen species (ROS) production by binding active redox metals (123, 127). However, the age-induced increase in ROS finally compromises the Zn-binding capacity of MTs (128). Similarly, Zn deficiency increases ROS and inhibits MT activity, resulting in compromised mitochondrial function and cytochrome c oxidase activity (129).

MTs can store and release Zn depending on cellular needs contributing to its homeostasis. The stored Zn is important for the rapid growth of the brain that occurs during the latter part of gestation. The localization of MTs in the brain was first described by Suzuki et al. (130), which identified that the onset of the system starts at about 21–22 weeks GA. MTs I and II also play a role also in the distribution of Zn in this phase as they are found in specific glial populations, located in the periventricular zones. These cells migrate towards the cortex starting at 21 weeks of GA till 35 weeks GA, with possible completion and maturation of the system up to 10th postnatal month (130, 131). The role of MTs in these cases, with clear co-localization with glial proteins might relate with processes that involve these populations such as myelin production and neuronal migration.

In Summary: MTs play a role not only as ROS scavengers and prevention of toxicity in the fetal brain but also maintain homeostasis of Zn and help match the energy and Zn demand in the developing brain and migrating glial cells .

### A6 insulin and glucose metabolism

Zn binds to insulin and contributes to its biosynthesis, crystallization, and post translational maturation (132, 133). Zn is transferred inside the insulin secretory granules of β-cells via the ZnT8 transporter. Lack of ZnT8 activity prevents insulin crystallization and secretion and may be associated with type 1 and 2 diabetes mellitus (DM) (134, 135). As mentioned in the previous section, Zn decreases the expression of pro-inflammatory cytokines of IL-1β, TNF-α, and IL-6. In Zn deficiency, the long-term activity of these cytokines results in apoptosis of β-pancreatic cells and insulin resistance.

Apart from insulin control, Zn also participates in glucose metabolism. By activating GLUT4 in cell plasma membranes, it facilitates the uptake of glucose by insulin-dependent tissues (136, 137). In addition, Zn can act as an insulin-mimetic, inhibiting forkhead box transcription factors (FOXO) and regulating necessary gluconeogenic enzymes (138). Lastly, Zn inhibits glucagon secretion by inhibiting voltage-gated channels in pancreatic α-cells (139–141).

The role of Zn concentration on hyper- or hypoglycemia is not well established in preterm newborns. Limited evidence from our group showed that after increasing dose of Zn in PN to recommended dose in 23–28-week GA neonates the number of hyperglycemic episodes decreased, without a change in hypoglycemic events (142, 143).

Finally, association of hyperglycemia and ROP (144, 145) could be explained by abnormal modulation of HIF-1 and VGEF, two key factors for the development of ROP which also dysregulate after hyperglycemic conditions (146–148).

In summary: Zn affects glucose metabolism through insulin maturation, GLUT4 activation and glucagon inhibition. Zn sufficiency could improve glucose metabolism, decrease hyperglycemia, and improve downstream abnormal signals related to ROP. These effects need further exploration.

### A7 superoxide dismutase

Superoxide dismutases (SODs) are crucial antioxidant enzymes that protect cells from ROS that arise in oxygen-rich environments from mitochondria, peroxisomes, and cytoplasm. SODs catalyze the conversion of superoxide to oxygen and hydrogen peroxide. Hydrogen peroxide is then eliminated by other antioxidant enzymes, such as catalase and glutathione peroxidases (149).

The catalytic properties of SODs depend on their metalation and disulfide bonding during posttranslational modifications (150). Zn and Cu are the catalytic metal ions for the human Cu-Zn-SOD (SOD1). The binding of the SOD1 homodimer to Zn offers structure stability, whereas Cu is responsible for enzymatic functionality (151). A stable connection with the His63 residue keeps Zn and Cu together and ensures the functionality of SOD1 even at extreme pH levels.

If SOD1 does not function normally, high ROS levels will cause oxidative damage, such as protein carbonylation, DNA breakage, and membrane lipid peroxidation (152). In adults, these can lead to different diseases, from cancer and amyotrophic lateral sclerosis to Parkinson's disease (153–155). Oxidative stress can also act as a stimulus for SOD1, so that it acts as a transcription factor. When hydrogen peroxide levels are high, SOD1 is phosphorylated via the cascade of Mec1, DNA Damage Response (DDR) kinase. The phosphorylated SOD1 then translocates to the nucleus, where, by binding to gene promoters, it controls the transcription of genes related to oxidative stress resistance (156).

SOD1 mutations can affect RNA metabolism. Mutant SOD1 can bind to the mRNAs of Neurofilament Light Chain (NFL) and Vascular Endothelial Growth Factor (VEGF) and form ribonucleoproteins complexes that then aggregate (157, 158). One of these RNA-binding proteins is HuR, which is protective against stress in motor neurons. Its interaction with the mutant SOD1 impairs its neuroprotective function and, along with the downregulation of VEGF, contributes to cytotoxicity and the appearance of neurodegenerative diseases like amyotrophic lateral sclerosis (159, 160).

In summary: Zn is the catalytic metal ion for SOD, a key antioxidant enzyme. If SOD is not adequate, high ROS levels can emerge resulting in oxidative stress, DNA breakage, lipid peroxidation and neuronal apoptosis.

### Part B Zn and the neonatal brain

Zn is abundant in various brain parts, especially the hippocampus, cerebral cortex, amygdala, olfactory bulb and retina (161). It plays crucial roles in synaptic transmission, neuronal signaling, and brain development in these regions. Zn deficiency or excess can result in abnormalities in neurodevelopment, behavior, CNS formation, and a variety of neurological diseases.

### B1 Zn in synaptic transmission and plasticity

**Figure 2:**
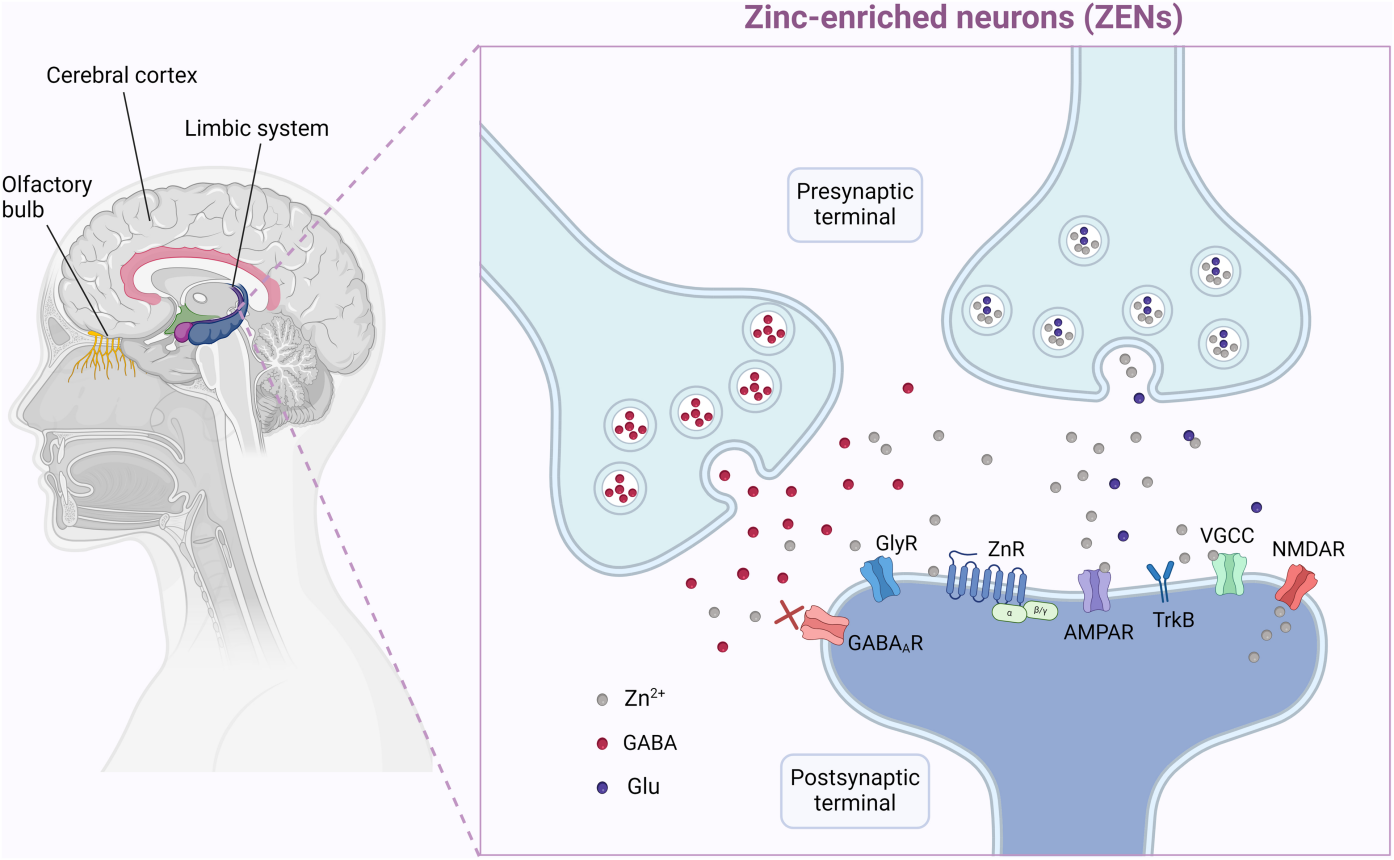
Zinc (Zn) exists in very high concentration is specific tissues and plays a key role in synaptogenesis, plasticity, neuronal repair and cellular migration and development. Here on the left we depict some of these such as the olfactory apparatus, the limbic system and cerebrum. These actions are mediated with specific Zn-enriched neurons (ZEN). ZENs contain high densities of the excitatory glutamate receptors, the N-methyl-D-aspartate (NMDA) and the α-amino-3-hydroxyl-5-methyl-4-isoxazole-propionate (AMPA), which have several modulatory Zn-binding sites. The NMDA and AMPA glutamate receptors regulate various calcium-, sodium-, and potassium-dependent intracellular pathways and play critical roles in brain function and development. In certain brain regions their function is greatly affected by the extracellular Zn levels. Created in BioRender. Chamakioti, M and Asimakopoulos, T. Abbreviations: (2024). AMPAR, α-amino-3-hydroxyl-5-methyl-4-isoxazole-propionate receptor; GABA (R), gamma-aminobutyric acid (receptor); Glu, glutamate; GlyR, glycine receptor; NMDAR, N-methyl-D-aspartate receptor; TrkR, tropomyosin receptor kinase receptor; VGCC, voltage-gated calcium channel; ZEN, zinc-enriched neuron; Zn, zinc; ZnR, zinc receptor. Created in BioRender.com. Asimakopoulos, T and Chamakioti, M (2024) Agreement Number MB26TVCEGU.

The hippocampus, which contains a high concentration of Zn, acts as the center of learning and memory and houses neuronal machinery responsible for stress responses. Accordingly, hippocampal Zn appears to be directly associated with learning, memory, and behavior (162, 163). Zn interacts with ligand-gated ion channels post-synaptically and modulates ion transport. Glutamatergic *Zn-enriched neurons* (ZENs) contain high densities of the excitatory glutamate receptors N-methyl-D-aspartate (NMDA) and alpha-amino-3-hydroxy-5-methyl-4-isoxazolepropionic acid (AMPA), which have several modulatory Zn-binding sites (163). ZENs are found in the hippocampus, the olfactory bulb, and the dorsal cochlear nucleus (162) (Figure 2).

The NMDA and AMPA glutamate receptors regulate various calcium-, sodium-, and potassium-dependent intracellular pathways. In certain brain regions, namely the neocortex, the amygdala, and the hippocampus, their function is greatly affected by the extracellular Zn concentrations (164). Zn has a dual effect and can bind differently to the glutamate receptors, attenuating or amplifying their signals, depending on their levels. Because NMDA receptors are pivotal in triggering long-term changes in synaptic efficacy, it is evident that Zn participates as a co-transmitter in cognition and plasticity of the synapses (128). Apart from glutamate receptors, Zn also interacts with gamma (γ)-aminobutyric acid (GABA) receptors (165), glycine receptors and metabotrophic Zn receptors (166–170).

Preclinical data show that gestational Zn deficiency in rodents caused significant alternation in the production of and expression of NMDA subunits, brain derived neurotrophic factor (BDNF) and nerve growth factor (NGF) which caused memory and learning impairments later in life (171).

In summary: Zn affects synaptogenesis, neuronal plasticity and synaptic efficacy and in these ways plays a role in memory and behavior .

### B2 Zn in brain development

Neurogenesis, the CNS development process, occurs during gestational and neonatal periods. During this period, the radial and tangential migration and the division of neural stem cells (NSCs) create the neural tube, the neural crest, and the notochord. More specifically, the invagination of the neural plate creates the neural tube, and this process is called neurulation (172). When the neural tube closes, the neuroepithelial cells of the ventricular zone become radial glial progenitor cells (RGCs), which later turn into glial cells and neurons (173).

### B3 Zn finger proteins (ZFPs) and neurogenesis

Zn, in the form of ZFPs, plays a crucial role in brain morphogenesis. ZFPs are involved in transcriptional regulation, signal transduction, actin targeting, and cell migration (96, 174). During neurogenesis, ZFPs direct the fate of embryonic stem cells (175). Zac1 protein participates in neuronal development in the cerebellum, Zn finger and BTB domain-containing protein 20 (Zbtb20) in the hippocampus, and fasciculation and elongation zeta protein (Fez) f1 and Fezf2 in the brain's olfactory region (176). ZFPs, e.g., Glioma-associated oncogene family Zn finger 3 (Gli3), regulate the cell cycle of RGCs, affecting their differentiation into neural cells. Interaction with the Sonic hedgehog protein (SHH) can alter the length of the G1 phase in the cell cycle of RGCs (153, 177). Mutations in Gli3 result in a shortened cell cycle, impaired cortical lamination, and cortical neuron formation and are responsible for various morphological CNS abnormalities, such as Greig cephalopolysyndactyly syndrome (GCPS), Pallister-Hall syndrome (PHS) and polydactyly and syndactyly (178).

Other characteristic examples are Zeb Zn fingers, Zeb1, and Zeb2. Zeb1 controls the development of the neocortex by acting as a transcriptional repressor that regulates the division mode of RGCs, their proliferation, migration, and differentiation. Zeb1 also affects the development of electrophysiological properties in developing neurons (179). Zeb2 participates in the differentiation of RGCs to Bergmann glial cells and astrocytes (180). Lastly, the Zic-type poly-ZNFs (Zic1, Zic2, Zic3, Zic4, and Zic5) take part in the closing of neural plates, the formation of the neural crests, the proliferation and the differentiation of NPCs in the medial forebrain, and cerebellar morphogenesis (181–183). Mutations in the Zic-type poly-ZNFs can hinder the physiological division of the brain into two hemispheres. They can also cause the Dandy-Walker malformation, which is associated with specific brain abnormalities such as cerebellar vermis hypoplasia and delayed motor and cognitive development (184).

### B4 the role of Zn in the different stages of neurogenesis

In short, neurogenesis can be divided into three main stages: NSC proliferation and migration, neuronal differentiation, and cell survival (185–188).

#### Zn in stem cell proliferation, glucocorticoid modulation and neuronal survival

Zn affects genes associated with the cell cycle, such as transcription factor AP-1, nuclear factor κB (NF-κB), and nuclear factor of activated T cells (NFAT) responsible for cell proliferation, differentiation, and apoptosis. This way, Zn controls the proliferation of neuronal precursor cells (189, 190).

Zn influences the levels of glucocorticoid hormones in the brain, and Zn deficiency increases their levels, resulting in numerous harmful effects on CNS development. Glucocorticoids induce extracellular accumulation of glutamate in hippocampal neurons, triggering excitotoxicity and cell death. Chronically increased glucocorticoid levels also damage the hippocampus via metabolic alterations that result in ischemia, hypoxia, and hypoglycemia and limited proliferation of NSC in the hippocampal dentate gyrus of primates (191–194). Finally, glucocorticoids interact with the Hedgehog signaling (HHS) pathway, as both low and high glucocorticoid levels result in the inhibition of HHS. This inhibition occurs via promotion of Notch/Hes-signaling and downstream inhibition of transforming growth factor (TGF) β- suppressor of Mothers against Decapentaplegic (SMAD)2/3-signaling respectively (195).

The p53 protein is a Zn-binding cell regulator and tumor suppressor. In Zn deficiency, p53 is translocated to the nucleus, resulting in cell cycle arrest (190). The extracellular signal-regulated kinase 1/2 (ERK1/2) pathway is down-regulated, hindering NSC proliferation. Zn deficiency during gestation affects neurogenesis by limiting the proliferation and differentiation of neurons in the fetal rat brain cortex and disrupting the cortical excitatory/inhibitory balance. This disrupts the formation of the physiological cortical structure, resulting in cognitive impairment (189, 196).

In summary: Zn directly affects the survival of primitive neurons by interfering with key intracellular processes and pathways such as transcription factors, glucocorticoids and the p53 system .

#### Neuronal differentiation

During early development, Zn plays an essential role in neuronal differentiation. It promotes the proliferation and differentiation of the pluripotent Adipose-derived mesenchymal stem cells (ADMSCs) into neurons. In the differentiated stem cells, Zn triggers neurite outgrowth, inactivates RhoA (a key guanosine triphosphate, GTPase), downregulates the ERK1/2 pathway, and promotes the expression of migration/neuronal genes such as the microtubule-associated proteins (MAP)2 and nestin (197). Lastly, it delays the radial migration of neurons during the cerebral cortex formation by elevating the glucocorticoid levels (198).

As expected, low Zn levels hinder neuronal differentiation during early development. Zn deficiency inhibits the dendritic differentiation of various cells, such as Purkinje and stellate cells, in the cerebellar cortex of 21-day-old rats and hinders the differentiation of human-induced pluripotent stem cells by modifying the Zn transporter gene expression (199–201).

TGFβ receptors (TGFβR) are thought to play a role in Zn regulation of neuronal differentiation. Zn deficiency impairs TGFβR subtype 2 induction after retinoic acid in human cell lines (202). TGFβR deficiency relates with neurodegenerative disorders, β amyloid peptide production and Alzheimer's disease (203). Zn deficiency reduces the expression of ZnT in young/multipotent neurons, increases cholinergic signaling among others and could change their developmental potential (202). Changes in cholinergic function of neurons has been shown in autism spectrum disorders (ASD) (204) and the role of Zn in these diseases processes has been previously reviewed (205).

In summary: These findings suggest that Zn is intrinsically involved in key pathways that affect neuronal differentiation such as TGFβ and cholinergic systems and its deficiency causes disruption of these pathways. Behavioral disorders such as ASD might be linked with these pathways.

#### Neuronal precursor survival

Zn also plays a role in the survival of neuronal precursor cells, as it regulates the expression of both pro-survival pathways, like the ERK, Ak strain transforming (Akt), and nuclear factor (NF)-kB pathways, and pro-apoptotic cascades, such as the Jun N-terminal kinase (JNK) and p53 pathways. In this context, Zn deficiency is responsible for altered neuronal differentiation, limited cell survival, and impaired synapse function (199). Low Zn levels are associated with activation of Caspase-3 and downregulation of the ERK pathway, reduced Ki67-positive cells, increased TUNEL-labeled cells in the subgranular zone (SGZ), arrest of the cell cycle in the G0 G1 phase, promotion of apoptosis in neurons, and modification of cell signals that control the pro-survival and pro-apoptotic gene expression, such as NF-κΒ and p53 (189).

#### Zn and gyration; cerebrum and cerebellar effects

The hippocampus and cerebellum are among the brain regions affected by Zn deficiency. Specifically, low Zn levels act in the cerebellar granular layer of rats and reduce the density of neurons, which also have shorter and less branched dendrites (200).

Zn deprivation significantly inhibits the development of the cerebellar cortex and delays the withdrawal of the external cell layer, the acquisition of granule cells, and the differentiation of Purkinje cells (200). It also impairs the dendritic growth of basket and stellate cells, interneurons of the cerebellar molecular layer that form and differentiate during the initial postnatal period (206, 207). The effects of Zn deficiency on the dendritic differentiation of these cells were studied in 21-day-old rats. It was shown that the total dendritic length of neurons and the dendritic field area of Zn-deficient animals were 43% and 30% smaller in the lower half of the molecular layer, respectively. This could be possibly explained by the delayed onset of dendritic differentiation and the slow rate of dendritic growth (200).

In summary: In preclinical studies, the hippocampus and cerebellum are affected when Zn deficiency is present. Specifically, the granule cells, and the differentiation of Purkinje cells are very sensitive to lack of Zn. These result in delayed dendritic development and cerebellar cortex abnormalities.

### B5 Zn and white matter development

Zn deficiency can affect multiple cell types in the brain.

#### Astrocytes

Zn deficiency during the early developmental period in rats downregulates the STAT3 signaling pathway, inhibiting astrogliogenesis and producing a low number of astrocyte cells in the early postnatal cortex (208). When these rats reach adulthood, the astrocyte number does not increase; it instead remains the same, representing an excellent example of the long-term consequences of Zn deficiency in the early stages of life (209). The homeostasis of Zn in astrocytes is maintained by a previously described Zn transporter system, with one subtype (Zn T1) to act as a protective mechanism against extreme accumulation of intracellular Zn (210).

#### Myelin production, oligodendrocytes and Zn

Zn is also critical for the development of oligodendrocytes (161, 211–214). Zfp488 is a ZFP specific for oligodendrocyte cells that co-regulates the gene expression during differentiation (215). Zn levels in developing oligodendrocytes remain high during differentiation and fall significantly after maturation is achieved, indicating a role of Zn in the differentiation of this cell type and a possible restorative potential of Zn for the failure of pre-oligodendrocyte maturation in premature infants with white matter injury (216, 217). Gestational deprivation of Zn has been shown to affect various myelin components in rodents and primates (218, 219).

In a severe but rare form of Zn deficiency in humans, as occurs in acrodermatitis enteropathica, several case reports have described cerebral atrophy, irritability, apathy, and psychomotor delay in the affected individuals (220, 221). On some occasions the findings are reversible upon Zn supplementation.

In summary: These effects show that Zn deficiency, especially if it occurs early in fetal development, can disrupt astrocytes, oligodendrocytes and myelin production, with potential lingering effects towards adulthood .

### B6 Zn and retina

Although Zn exists in many ocular tissues, such as the choroid, ciliary body, and iris, the highest Zn concentrations in the eye are found in the retina-choroid complex (464–472 μg/g Zn of dry weight) (222). Zn is an element necessary for many ocular metalloenzymes, both from a structural and functional aspect. Deficient Zn status can hinder ocular development, especially during the early prenatal stages (222). In the eye, vitamin A is converted to its active form, retinal, by a Zn metalloenzyme, alcohol dehydrogenase (ADH). Retinal is needed for the synthesis of rhodopsin, the photopigment found in the retinal rods responsible for night vision. Therefore, depressed ADH activity in cases of Zn deficiency would result in night blindness (222). In pregnant women with nyctalopia due to combined vitamin A and Zn deficiency responded to vitamin A and Zn supplementation but not to vitamin A or Zn alone (223). In addition, Zn has an important protective role against glutamate-induced toxicity in the retina. The retina is abundant in glutamatergic neurons. Zn is released along with glutamate in the retina, just like in the brain. By binding on the NMDA glutamate receptors of retinal ganglion cells and blocking excitation, Zn prevents the toxic effects of glutamate on the retina (224).

In preterm infants, the main concern is the possible association of Zn deficiency with the development of ROP. The pathophysiology of ROP evolves in critical hyperoxic and hypoxic phases with key enzymatic pathways such as VEGF, erythropoietin (EPO) and IGF-1 among others to play important roles in each phase (225–227). The role of Zn in the development of ROP is controversial and not well studied but could affect the development of ROP by several mechanisms: (1) as a potent antioxidant; (2) as modulator of glutamate receptors and amelioration of related toxicity as noted above, especially since glutamate can induce VEGF, a key molecule for development of ROP (228); (3) via modulation of transcription factors. Zn has antioxidant properties, as it is part of certain protective antioxidant enzymes, such as the previously mentioned enzyme SOD1, which has a protective effect against oxygen-induced retinopathy in mice (229). However, preterm neonates often have deficient Zn levels and subsequent reduced SOD activity. Moreover, Li et al. recently identified through a genome wide association study a novel lead single nucleotide polymorphism (SNP) that fell in an intronic region within the GLi3 gene. GLi3 is critical for the differentiation of the retinal pigment epithelium (RPE) and rod photoreceptor layer, functioning as both a transcriptional activator and repressor of canonical SHH signaling (230, 231). It also controls both the innate and adaptive immune response (232, 233), and, as aberrant inflammation is involved in the pathophysiology of ROP (234, 235), there is a possible association between GLi3 and ROP. The role of Zn related to EPO is quite complex, and to our knowledge indirect, and will be discussed briefly in the next paragraph. Clinically, exogenous human EPO initiated shortly after birth, although could result in angiogenesis, was not found to increase the risk of severe ROP (Preterm Erythropoietin Neuroprotection Trial, PENUT) (236).

Several clinical observational studies in premature newborns attempted to investigate the role of Zn with ROP, with so far mixed results. In one study, preterm newborns with ROP had lower maternal Zn levels, cord levels and serum levels at 40 weeks PMA when compared to those who did not develop ROP (237). In a retrospective study that involved infants 28–37 weeks GA, investigators measured one serum Zn level at less than 24 h after birth and in those newborns with levels <0.7 μg/ml, a higher proportion developed ROP when compared to those with higher Zn level (42% vs. 24%, *P* = 0.02) (59). However, in a systematic review of RCTs, enteral Zn supplementation compared to no supplementation or placebo had no effect on ROP (238, 239). Notably, several of the included studies also involved infants >32 weeks GA, with low baseline risk of ROP. The efficacy of maternal Zn supplementation for the prevention of ROP remains to be investigated.

In summary: Preclinical data link Zn deficiency with the development of ROP and other developmental eye disorders. Specifically, Zn could improve ROP via its antioxidant properties, modulation of glutamate pathway and excitotoxicity and via changes of downstream transcription factors. Nevertheless, robust clinical data to support this mechanistic link, is lacking so far.

### B7 Zn and Vitamin D

Vitamin D is a lipid soluble vitamin with a steroid structure. Its structural integrity and functions are tightly regulated by Zn. Cholecalciferol is hydroxylated in the liver into 25-hydroxycholecalciferol, which is further hydroxylated in the kidney into 1,25-dihydroxycholecalciferol (active form). The latter interacts with vitamin D receptors (VDR). Once vitamin D interacts with its receptor, VDR dimerizes with the retinoid X receptor (RXR), to upregulate several downstream genes, including the calcium stimulated ATPase and alkaline phosphatase, causing increased intestinal absorption of calcium. VDR uses Zn to regulate the actions of vitamin D-dependent genes. *In vitro,* in the absence of Zn, VDR conformation cannot occur, and vitamin D function fails (240, 241). Zn supplementation increases Vit D levels and there a positive correlation between vitamin D levels and Zn levels (242, 243). Low Zn levels were found to predict vitamin D deficiency in adolescent women (244). On the other hand, vitamin D regulates Zn homeostasis by affecting the expression of Zn transporters (245). The role of vitamin D in normal brain function and development has been reviewed and demonstrated in several studies (246–248).

In summary: The intrinsic role of vitamin D in the regulation of Zn and vice versa should be taken into consideration when interpreting studies with Zn deficiency .

### B8 Zn and factors that regulate hemopoiesis—possible roles in neuronal development

Zn participates in hemopoiesis in the form of ZFPs. Different ZFPs act on different cell lineages and maturation stages. ZFPs like GATA, Ikaros, FOG-1, Snail, MOZ, Gfi, and Zfp521 regulate the survival and differentiation of hematopoietic cells at the stem cell level until they commit to the lymphoid or myeloid lineage (249). Lymphopoiesis is regulated mainly by the ZFPs of the Ikaros family while myelopoiesis is controlled by a more complicated and diverse expression of ZFPs and has not yet been thoroughly studied. Among the 6 members of GATA-binding transcription factors of ZFPs, GATA-1 is important for erythroid cell line development (249). Zn is essential for lymphopoiesis and myelopoiesis, and it is also very important for erythropoiesis, mainly through the previously mentioned transcription factor GATA-1, the apoptotic proteins such as caspase 3, and the hypoxia-inducible factor (HIF) (250).

In the stage of proerythroblasts, GATA-1 release is triggered by the binding of erythropoietin to the erythropoietin receptor. Then GATA interacts with its co-factor, friend of GATA protein 1 (FOG-1), and together as a complex, they control gene expression (251). The formation of the complex requires the presence of Zn (252, 253). In addition, Zn, along with carnitine, inhibits the cleavage of caspase 3, a key component of apoptosis that is expressed when stimulation by erythropoietin is interrupted (254, 255). In this way, Zn prevents RBC apoptosis and promotes RBC survival, whereas Zn depletion results in RBC death (256, 257).

Erythropoiesis is initiated in hypoxic states when the HIF triggers the expression of the EPO gene. When oxygen levels are low, the HIF-1 subunit becomes stabilized and assembled. In contrast, in physiologic oxygen levels, HIF-1 is degraded. An HIF-1-specific prolyl-hydroxylase (PHD) hydroxylases proline-564 and/or −402 residues of HIF-1, signaling its ubiquitination and degradation by the proteasome. PHD2, also known as EGLN1, the main enzyme in control of hydroxylating HIF-1, has a conserved catalytic domain similar to that of other prolyl-4-hydroxylases. However, it also has a distinctive N-terminal MYND-type Zn finger domain (258) that has been anticipated to have either a positive or a negative regulatory function. Varying roles of this domain have been indicated, and according to the most recent results from Sinnema et al., the Zn finger ordinarily has a positive regulatory effect on the catalytic activity of PHD2. Specifically, a human PHD2 Zn finger mutation results in a loss of PHD2 function and is associated with congenital erythrocytosis (259).

HIF not only has functional roles but also takes part in the morphogenesis and development of the CNS. As shown by preclinical experiments, brain angiogenesis depends on the HIF signaling pathway since new vessel development is affected by oxygen regulation in neurons (260). In addition, HIF has a significant role in the neurogenesis of the autonomic/sympathetic nervous system (ANS), as the loss of HIF-1α hindered the survival and proliferation of pre- and post-ganglionic neurons. Specifically, lack of HIF-1α in the cardiac outflow tract, right ventricle and atrium, pharyngeal mesoderm, peripheral neurons, and hindlimbs caused hypoplasia of the sympathetic ganglion chain and diminished chromaffin cell number in the adrenal medulla (261). Astrocytes interact with HIF-1a in the opposite way; the deletion of HIF-1α prevents a hypoxia-induced cell death (262). Interestingly, the deletion of HIF-1α in neurons facilitates their hypoxia-induced cell death (262). Finally, HIF can induce hypomyelination by inhibiting the differentiation of oligodendrocyte precursor cells (263, 264).

In summary: The role of Zn in factors that affect hemopoiesis and lymphopoiesis is well supported by preclinical studies. HIF-1 is a regulator of erythropoiesis under conditions of hypoxia, but also a key regulator of brain morphogenesis, astrocyte function, myelination and vascular development of the CNS and ANS. Specific Zn containing enzymes (Zn-fingers) affect HIF-1 function, but whether Zn deficiency has significant effects on those pathways remain to be investigated.

### Part C clinical aspects of Zn provision

#### C1 can Zn induce neuronal toxicity?

Zn reaches physiologically high but transient concentrations in synaptic cleft. The synaptic clefts are enclosed low volume cylindrical compartments which represent only 1% of the extracellular fluid (ECF) of the brain (265). In these areas, Zn concentration is tightly regulated by receptors and local MTs (type III) (266) and is estimated to be 1–100 μM (267).

Zn is released and possibly reaches toxic levels during pathological events such as cerebral ischemia or seizures. Nolte et al. assessed the effect of extracellular Zn on cultured astrocytes *in vitro* and found that Zn at concentration of 200–250 μM induced cell death within 1.5–2 h of exposure (210). The efflux of Zn after noxious stimuli in a confined ECF space may alter the pH of the microenvironment, leading to cellular damage, with subfield CA3 hippocampal neurons being very sensitive to early destruction (268–270). Induction of convulsions with kainic acid (KA) in rats can induce an increase in serum and ECF Zn but overall depletion of tissue Zn (271), while Zn appeared to accelerate brain infarction after focal ischemia in rats (272).

During global brain ischemia, Zn might become toxic and result in selective neuronal loss independently of other mechanisms of brain damage such as excitotoxicity. In rats, after brain ischemia, Zn accumulated in the hippocampal hilus and CA1, as well as in the cerebral cortex, thalamus, striatum, and amygdala, while Zn chelation showed reduced ischemic neuronal degeneration (273).

High neuronal synaptic Zn concentrations (up to 300 μM) are present transiently in specific areas in rodent CNS (such as the hippocampal mossy fibers) after neuronal hyperexcitation with kainic acid (KA), a process that is calcium mediated and might have a role in physiologic neuronal excitation (267, 274). Lower Zn concentrations (3–30 μM) in the synaptic cleft of cultured rat retinal neurons, was sufficient to protect from the toxicity of glutamate or NMDA (224).

Zn neurotoxicity might be associated with the proto-oncogene c-Src (abbreviation for cellular sarcoma), a non-receptor ubiquitous cellular tyrosine kinase. Src kinase is an essential regulator of cellular physiological processes ranging from differentiation, mitogenic signaling to motility and neuroinflammation (275–277). The brain expresses 200-fold higher levels of this protein than most other cells (276). Zn is released along with glutamate in the CNS and inhibits NMDA receptor activation. Following this blockade, use of high (but sublethal) concentrations of Zn in rodent cortex can cause a Src kinase-mediated up-regulation of NMDA receptor activity and subsequent cytotoxicity (278).

Inflammation could also contribute to Zn toxicity, since high extracellular Zn concentrations may be pro-inflammatory in primary mononuclear cells. In a study of human PBMCs where high concentrations of Zn (>100 μM) were achieved, all types of cytokines were increased, and pro-apoptotic genes were induced (279).

The above studies show that Zn has the potential to induce neuronal toxicity especially after pathologic conditions (seizures, stroke and ischemia), either directly or indirectly via excitotoxicity or/and inflammation. High Zn extracellular concentrations can be physiologic, with trophic and receptor modulatory properties in specific neurons and not related to toxicity. Of note Zn concentrations of 100 μM (μmoles L) correspond to 6.53 μg/ml—although these levels are reported in the ECF or synaptic cleft of CNS and cannot be compared directly with serum levels, they are much higher than the upper normative serum Zn level that was previously discussed (1.43 μg/ml) and 100 times more than Zn in CSF.

#### Indirect Zn toxicity

While Zn may interact with many elements, attention has been drawn to its relationship with Fe, Cu, calcium absorption, vitamin D and vitamin A. Even though Zn supplementation is considered relatively safe, enteral administration has the potential to negatively influence Cu and Fe absorption in the GI tract (69, 280, 281). By competing for the same receptors, Zn reduces Cu uptake (2, 282–285). Although neonatal data are lacking, in adults a dose of Zn: Fe of 5:1, significantly decreased Fe absorption (286, 287). Limited data in rats show that Zn could interfere with Vitamin A, by decreasing its liver mobilization and release, whereas similar studies in neonates are lacking (288). Therefore, Zn supplementation over and above the recommended daily intake requires careful monitoring and evaluation for patients dependent on a balanced micronutrient intake. Although definition of “prolonged” Zn provision is not well defined, any newborn that has received >2 weeks of Zn provision could be at risk for Cu deficiency unless Cu is supplemented at a 10:1 Zn: Cu weight ratio and at alternate times of administration. A recent systematic review of RCTs in children 6 months-6 years demonstrated a small decrease in Cu concentration in Zn-supplemented patients when compared to no Zn. This result of unclear clinical relevance was based on studies that provided prolonged Zn supplementation with no supplemented Cu (289).

In summary: Direct neuronal Zn toxicity is unlikely to occur under physiologic conditions with routine Zn supplementation but could be encountered under conditions of cerebral ischemia, stroke, or seizures. In these circumstances, Zn supplementation needs to be held. Indirect Zn toxicity, secondary to its effect on Cu and Fe absorption needs to be carefully monitored especially in an era of increasing provision of Zn. Adequately powered studies that will investigate the potential toxicity of Zn provision in premature neonates, given the trends towards higher dosing supplementation, are urgently needed.

### C2 alcohol fetal exposure, fetal alcohol syndrome (FAS) and Zn

Alcohol consumption may interfere with Zn tissue utilization and can have significant effects on the fetus (290, 291). Alcohol consumption can result in deficiency of several micronutrients including Zn. Whether Zn deficiency can contribute to the development of fetal alcohol syndrome (FAS) - a devastating disease with serious neurodevelopmental effects- is controversial (292). Zn is part of ADH, which detoxifies alcohol and generates aldehydes and ketones as metabolites. Alcohol in high concentrations can deactivate this enzyme (293). In addition, alcohol can induce increased urinary Zn excretion and low plasma levels in pregnant women (294), while alcohol abstinence reverses zincuria in adults (295). Affected infants from FAS could also present with low plasma and high urinary Zn excretion (296). In alcohol-exposed primates, when adequate Zn (3.5 mg/day) was given, maternal and neonatal brain Zn concentrations were similar to non-exposed controls, indicating that placental Zn transfer was not significantly affected (297). In rodents, whether alcohol affects Zn placental transfer is controversial (298, 299) and Zn supplementation might not be able to restore Zn transplacental movement in alcohol exposed mothers (300). In addition, ethanol-exposed rats had similar Zn placental transfer when compared to non-ethanol fed controls (301).

In summary: Alcohol consumption could be associated with multinutrient and/or isolated Zn deficiency. Although some of the manifestations of FAS may be related to co-existent or alcohol induced Zn deficiency, further research is needed to identify its significance in brain outcomes in the affected neonates.

### C3 smoking, Zn and the role of Cadmium (Cd)

The effects of smoking during pregnancy on fetal Zn are largely mediated by the Cd content of cigarettes. Cd and Zn are closely related in many metabolic pathways and often antagonize one another in their usage. Cd strongly induces MTs, which bind both Cd and Zn and can lower systemic Zn levels (302, 303). Smoking during pregnancy increases Cd levels in maternal blood and in placenta (302, 303). However, a large quantity of Cd is sequestered in the maternal liver and kidneys, with only a fraction accumulating in the placenta and the fetus (303). Mouse models have shown an increase in MT in the placenta after MT elevations in the liver have taken place, which tends to sequester Zn in the process, decreasing delivery of Zn to the fetus (52).

Additionally, cord blood analyses have shown that in mothers that do not smoke, elevations in placental Zn are associated with elevations in cord Zn, whereas, in mothers who smoke, elevations in placental Zn are not accompanied by elevations in cord Zn (302). Another notable feature of Cd's effect on maternal-fetal Zn delivery is through Zn transporters. In mouse models, Cd downregulates Zn transporters, which possibly could decrease Zn fetal uptake (303, 304). Mouse models have further associated maternal Cd exposure with a decreased BDNF and Zn level in the brain (304), indicating that smoking during pregnancy may impact fetal brain development. Supplementation of Zn appears to restore BDNF levels in fetuses (304).

In summary: Smoking affects Zn levels in the fetus primarily via Cd-mediated upregulation of MTs, and downregulation of Zn transporters, which together, act to sequester Zn in placental tissue and can harm fetal brain development as a downstream effect.

### C4 clinical data in premature neonates, infants and children—neurodevelopmental outcomes and Zn

The neurodevelopmental effects of Zn in preterm newborns can be related to (1) its deficiency (low Zn level) (2) to its adequate supplementation or (3) to indirect effects on growth. Systematic reviews have shown that Zn supplementation increases growth and decreases mortality in preterm infants (238, 239). These findings are summarized in a Cochrane review (238) including one study with high-dose Zn intake [(305), ∼10 mg/day] and 4 studies with low (currently recommended) dose were included (63, 64, 306, 307). Other studies with high-dose Zn intake [(308, 309, 310) were not included]. The included studies were relatively small but had good methodological quality. The meta-analysis showed that enteral Zn supplementation may decrease all-cause mortality [Relative Risk (RR) 0.55, 95% Confidence Intervals (CI) 0.31–0.97; 3 studies, 345 infants; low-certainty evidence based on high-dose Zn study by Terrin et al. (305)], while had little or no effect on common morbidities such as bronchopulmonary dysplasia, ROP, bacterial sepsis, or NEC (low certainty of evidence). The authors concluded that Zn supplementation probably improves weight gain [standardized mean difference (SMD) 0.46, 95% CI 0.28–0.64; 5 studies, 481 infants; moderate-certainty evidence]; and may slightly improve linear growth (SMD 0.75, 95% CI 0.36–1.14, 3 studies, 289 infants; low-certainty evidence), but had minimal effect on fronto-occipital head circumference (FOC) (SMD 0.21, 95% CI −0.02 to 0.44, 3 studies, 289 infants; moderate-certainty evidence). No data was available on long-term neurodevelopmental outcomes at 18–24 months of age. In a systematic review that focused on anthropometrics and neurodevelopment only, Alshaikh et al. (239), included 8 RCTs studies (63, 64, 305–308, 311, 312) two of which utilized high dose (∼10 mg/day) Zn (305, 308). 7 out of 8 RCTs (742 infants), reported growth data at 3–6 months corrected age and 2 reported neurodevelopmental outcomes at 6–12 months, and will be further discussed later in this chapter (306, 312). Zn supplementation was associated with increased weight z-score (SMD 0.50; 95% CI 0.23–0.76), length z-score (SMD 1.12; 95% CI 0.63–1.61) and motor developmental score (SMD 9.54; 95% CI 6.6–12.4). There was no effect of Zn supplementation on FOC.

Most of the studies reported in both systematic reviews included older premature and low birth infants and as result, outcomes that occur more frequently in very low birth weight (VLBW) and extremely low birth weight (ELBW) infants and very preterm (28–32 weeks or extremely preterm (<28 weeks) infants (such as ROP, NEC etc.) were not assessed adequately.

Our group showed that In Zn-deficient ELBW preterm newborns, low dose Zn provision for at least 2 weeks improved the *Z*-score of FOC, but not length and weight (58). In another study in Japan, routine enteral Zn supplementation at 3 mg/kg/day, starting at 2 weeks of life to discharge without any PN Zn intake, showed no effect on growth parameters (313). In contrast, higher and incremental enteral doses of Zn (5–10 mg/day) in VLBW infants, improved linear growth in a study from India (314).

Differences in Zn dosing, duration of treatment and initiation and differences in baseline maternal Zn deficiency could all contribute to the variation in the above outcomes. Overall, though, Zn provision in premature infants has been shown to improve growth quite consistently (238, 239).

Isolated or balanced postnatal growth patterns in various parameters [FOC, length, weight, growth velocity and/or body mass index (BMI)] are all associated, and possibly independently, with improved neurodevelopmental outcomes in preterm infants (315–318). From those growth parameters, isolated linear growth in VLBW infants is associated with improved language scores in the Bayley-III scores and less chance for future developmental deficits (319, 320). Improved anti-inflammatory milieu and glucose-regulatory hormones (321–323) are important factors for adequate growth in preterm newborns. Zn provision could relate via its well-described anti-inflammatory and glucose regulatory properties towards this effect (148).

In summary: Several studies have shown consistently improved weight gain and linear growth in preterm newborns even after low or moderate (as currently recommended) Zn supplementation dosing schedules. Since normal growth can be independently associated with improved neurodevelopment, and Zn improves growth, it needs to be further investigated if and to what extent Zn supplementation can contribute or mediate a potential independent positive effect towards neurodevelopment.

So far, the direct link between Zn provision and neurodevelopment in newborns is not well established, and most data are derived from older infants and children. The overall effect of Zn supplementation in infants or children on neurodevelopmental outcomes is not convincing, possibly since brain development and growth is less dependent on Zn later in life.

Clinical data in infants and children: In infants, 1–12 months of age, Zn intake improves length and weight (324, 325) while higher Zn dose (10 mg/day vs. 5 mg/day) had a higher impact on those growth parameters (325). The effect of Zn supplementation—especially when administered alone- was investigated in a systematic review of 96 studies in 219,584 children (age: 6 months to 6 years) and showed benefits in Zn status, diarrhea- related morbidity, linear growth and possibly a small positive impact on all-cause mortality (289). Regarding neurodevelopment, Zn supplementation was found to improve minimally the executive function and motor development (326) or have no global effect (327), while supplementing mothers during pregnancy was not found to improve long term developmental outcomes (324, 325). On the other hand, Zn supplementation in infants who reside in areas with high baseline Zn deficiency, improved cognitive and sensorimotor developmental outcomes (328). A systematic review that included children 0–5 years (25 studies and 11,559 patients), showed no significant efficacy of Zn with and without Fe co-supplementation on child mental and motor development up to 9 years old age (329). Finally, a systematic review in children with attention-deficit/hyperactivity disorder (ADHD) has shown that Zn supplementation may improve total ADHD scores (330).

In summary: These studies in older infants and children show that the effects of Zn supplementation on neurodevelopmental outcomes are minimal and possibly more pronounced in individuals with baseline Zn deficiency .

In newborns, only a few RCTs have reported limited neurodevelopmental outcomes (Table 1). Of them, three reported neurodevelopmental outcomes at 6–12 months prematurity-corrected age and one at ≤3 months.

**Table 1:** Neonatal randomized controlled studies (RCT) that assessed neurodevelopment with zinc provision.

Author	Country	N (both groups)	GA (weeks), Mean ± SD	BW (grams), Mean ± SD	Duration of supplementation	Dose of Zn	Neurodevelopmental outcomes	Age of assessment
Ragab et al.	Egypt	80	34.10 ± 1.24 weeks vs. 34.10 ± 1.24 weeks,	2,188.9 ± 203 vs. 2,186.3 ± 202.3	Day 1 of life till 6 months	2 vs. 0 mg/kg/day	ASQ Zn better than placebo	4 and 6 months
Friel et al. ^a^	Canada	50	29 ± 2.9 (mean initial total cohort) At R: 37 ± 1 vs. 36 ± 0.5	1,117 ± 289 At R: 1,842 ± 116 vs. 1,867 ± 100	1 month prior to discharge were randomized and received different formulas up to 5 months	2.2 mg/kg/day vs. 1.2 mg/kg/day (initial), decreasing with time—after 6 months equal Zn dose	Griffiths scales Zn better than placebo in motor scores, but not in total scores	3, 6, 9 and 12 months
Mathur et al.	India	100	33.5 ± 2.2 vs. 33.4 ± 2.3	1,603.7 ± 452 vs. 1,630.8 ± 479	7 days of life till up to 3 months corrected age	2 vs. 0 mg/kg/day	Amiel-Tison score Zn better (Alertness, hyper-excitability, Bicipital reflex and patellar reflex)	40 weeks corrected age and at 3 months

Values represent Zn supplementation group vs. controls.

Abbreviations: ASQ, ages and stages questionnaire; BW, birthweight; GA, gestational age; LR, at time of randomization; SD, standard deviation; vs., vs.; Zn, zinc.

^a^ Friel et al. Zn group received formula and supplemented zinc-copper drops (final concentrations: Zn group, 9 mg/L of Zn and 0.9 mg/L of copper vs. Placebo, 6.7 mg/L Zn and 0.6 mg/L of copper). The maximum supplementation of Zn per weight was at randomization and shown at the table.

Ragab et al. (312) in a study completed in Egypt, used the parents-completed Ages and Stages Questionnaires (ASQ) at 4 and 6 months of life and showed improvement in communication, gross and fine motor skills, problem solving and social interaction in infants who received Zn supplementation. Friel et al. (306), conducted a study in the US in 1993 and reported a significant increase in motor developmental score using Griffiths mental development scale using Amiel-Tison neurologic assessment at 3, 6, 9 and 12 months of life (331). Mathur et al. (64), conducted a study in India and found that Zn provision improved alertness and attention pattern at 40 weeks post menstrual age and decreased hyper-excitability at 3 months corrected GA. As mentioned earlier, two of these studies (64, 306) were evaluated in a systematic review by Alshaikh et al. who showed an improvement in motor developmental scores, but not in global development (239).

In summary: The above-mentioned studies overall show benefits in early neurodevelopmental outcomes after Zn provision, although the time frame of observation is up to 12 months after birth, which does not meet current standard for follow-up to 22–28 months corrected age or school age .

Whether Zn levels could be associated with poor developmental outcomes is also poorly investigated. Terrin et al. (37) showed that in preterm newborns 23–34 weeks GA, there was a significantly positive correlation between total composite motor score (with Bayley III scale) and serum Zn levels at 28 days of life (DOL) (*R* = 0.467, *P* < 0.05) (33758400). The same authors found that serum Zn levels at 28 DOL were dependent on energy (*β* −0.650; *P* < 0.001) and protein (*β* −0.669; *P* < 0.001) intake received through PN in the first week of life.

In summary: Currently there is limited evidence to suggest low Zn levels (early, or later in life) relate to poor neurodevelopment. The interaction of Zn levels, energy consumption and neurodevelopment need further research.

### C5 optimal dosing in premature neonates

Recent nutritional recommendations favored an increase in daily Zn supplementation (enteral Zn 2–3 mg/kg/day, parenteral Zn 500 μg/kg/day) (1–3). The enteral recommendation might not match physiologic data of transplacental fetal Zn accretion and actual needs for Zn might not be met, especially given the limited amount of Zn absorbed in the premature gut (32, 37). In addition, current recommendations, do not suggest different dosing schedules in cases of maternal Zn deficiency as commonly encountered in mothers with high poverty index and in women living in or recently immigrated from countries with endemic Zn deficiency (17). High Zn dosing (10 mg/dose) has been implemented by some investigators (305, 308, 332), but no neurodevelopmental outcomes have been provided so far. These studies have not reported major toxicity related to Zn and some have reported improved early outcomes, e.g., mortality, sepsis, necrotizing enterocolitis, feeding tolerance and/or growth. In Table 2 we summarize possible gaps of knowledge and future research directions.

**Table 2:** Gaps of knowledge and future research directions in Zn and neonates.

Type of uncertainty	Method for resolution
• Optimal enteral Zn dosing in VLBW VPT neonates is not known • Most studies use 2.5–3.5 mg/kg/day of Zn	• Conduct RCT with high (>5 mg/kg/day or 10 mg/day) compared to currently recommended dosing
• Optimal timing of initiation of enteral Zn is not known • Most studies start when infant is on full feeds • Limited data suggest early (1st week of life) could be beneficial	• Conduct RCTs that include early Zn protocol for infants not receiving PN
• Description of normative Zn levels based on GA and PMA • Given that neonatal serum Zn levels decrease with GA (and PMA), fixed normative levels as currently suggested (0.74–1.46 μg/ml), might need to be revised	• Conduct cohort studies with assessment of serial Zn levels and their relationships with outcomes • Optimization of Zn normative definition
• Role of maternal Zn levels and definition of fetal (early Zn) deficiency • Individualization of Zn supplementation based on maternal Zn status	• Assessment of maternal Zn status and correlation with neonatal serum levels and morbidity • Cohort studies could be nested in RCTs
• Systematic reviews involving Zn are lacking in two areas: - include studies with high-dose Zn - focus on VLBW-VPT neonates that have higher incidence of early neonatal morbidities (ROP, NEC etc.)	• Conduct systematic review to include studies with high dose Zn
• Short term brain effects of Zn deficiency and Zn supplementation on neonatal brain are lacking	• Conduct well organized prospective (or retrospective) cohorts to include head ultrasound and brain MRI findings (e.g., white matter injury) at term corrected age
• Assess long-term neurodevelopment after Zn supplementation and/or after Zn deficiency	• Conduct well designed RCTs and observational cohorts, able to assess long-term NDI at corrected age of 22 months in preterm infants
• Effects of Zn on biochemical factors (mechanisms)	• Assess Vitamin D, inflammation etc. • Correlate with disease processes

Abbreviations: GA, gestational age; MRI, magnetic resonance imaging; NEC, necrotizing enterocolitis; NDI, neurodevelopmental impairment; PMA, postmenstrual age; RCT, randomized controlled trials; ROP, retinopathy of prematurity; VLBW, very low birth weight, VPT, very preterm neonates; Zn, zinc.

In summary: We believe that well organized RCTs that include early Zn provision, at different - possibly higher - dosing schedules and include adequate neurodevelopmental follow up, need to be undertaken as soon as possible, in low-, middle- and high-income countries .

## Conclusions

In conclusion, Zn as a micronutrient, has a variety of physiologic roles that affect brain function and autoregulation but also in neuronal growth, migration and survival. Preterm newborns lose the period of highest fetal Zn accretion—the last two trimesters—and as result represent a population that is at very high risk for Zn deficiency. Additional risk factors, such as maternal Zn deficiency can exacerbate neonatal Zn deficiency, while feeding practices—such as exclusive unfortified DHM - can also result Zn deficiency.

Despite the emerging role of Zn as a key nutritional supplement, there are only a few, well organized published studies that investigate its role in short term neonatal morbidities and long-term neurodevelopmental outcomes. We suggest that multicenter RCTs be undertaken to investigate the above issues.
